# A real‐world study of dacomitinib in later‐line settings for advanced non‐small cell lung cancer patients harboring *EGFR* mutations

**DOI:** 10.1002/cam4.4495

**Published:** 2022-01-12

**Authors:** Hong‐Shuai Li, Jin‐Yao Zhang, Xiang Yan, Hai‐Yan Xu, Xue‐Zhi Hao, Pu‐Yuan Xing, Yan Wang

**Affiliations:** ^1^ Department of Medical Oncology, National Cancer Center/National Clinical Research Center for Cancer/Cancer Hospital Chinese Academy of Medical Sciences and Peking Union Medical College Beijing China; ^2^ Department of Oncology, The Fifth Medical Center Chinese PLA General Hospital Beijing China

**Keywords:** application, dacomitinib, later‐line, non‐small cell lung cancer, real‐world

## Abstract

**Objective:**

Dacomitinib has been approved for the first‐line treatment of non‐small cell lung cancer (NSCLC) carrying classical epidermal growth factor receptor (*EGFR*) mutations; however, real‐world data on its later‐line application are lacking.

**Materials and methods:**

Patients’ data were retrospectively collected from the Chinese National Cancer Center and the PLA hospital between August 2019 and August 2021. Kaplan‐Meier method and Log‐rank test were utilized to assess progression‐free survival (PFS) and overall survival (OS). Univariate and multivariate Cox regression analysis was conducted to determine prognostic indicators.

**Results:**

In total, 56 NSCLC patients harboring *EGFR* mutations treated with later‐line single dacomitinib or combinatory dacomitinib were enrolled. A total of 53 patients (94.6%) had treatment‐related adverse events; eight patients (14.3%) had grade 3 or 4 events. Among 49 evaluable patients, 26.5% (13 patients) had a confirmed partial response and 73.5% (36 patients) had disease control; the median duration of follow‐up was 9.6 months (95% confidence interval [CI], 8.4–10.8 months), the median progression‐free survival was 5.4 months (95% CI, 3.5–7.3 months), and the half‐year, 1‐year, and 2‐year OS rate were 79.2%, 70.6%, and 64.1%, respectively. Univariate analysis suggested that smoking, line of dacomitinib, and interval between last *EGFR*‐tyrosine kinase inhibitor (TKI) and dacomitinib were associated with PFS and OS; chemotherapy between last *EGFR*‐TKI and dacomitinib, and *EGFR*‐TKI generation followed by dacomitinib were respectively associated with PFS and OS; multivariate analysis indicated chemotherapy between last *EGFR*‐TKI and dacomitinib negatively affect PFS, and smoking and third‐generation *EGFR*‐TKI followed by dacomitinib negatively affect OS.

**Conclusions:**

This real‐world study has shown that dacomitinib is active and well‐tolerated in NSCLC patients harboring different *EGFR* mutations in later‐line settings, even for those with brain metastases. Patients who benefited more from the first TKI were more likely to benefit from dacomitinib, and earlier application of dacomitinib after front‐line TKI resistance may be considered.

## Graphical abstract

This real‐world study has shown that dacomitinib is active and well‐tolerated in NSCLC patients harboring different EGFR mutations in later‐line settings, even for those with brain metastases. Patients who benefited more from the first TKI were more likely to benefit from dacomitinib, and earlier application of dacomitinib after front‐line TKI resistance was suggested.

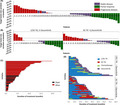

## 1 INTRODUCTION

Dacomitinib is a second‐generation (2G), irreversible, highly selective epidermal growth factor receptor‐tyrosine kinase inhibitor (*EGFR*‐TKI) that inhibits signaling from both heterodimers and homodimers of all members of the human *EGFR* family. The glorious ARCHER 1050 trial laid the foundation for its use as one of the standard first‐line options in patients with *EGFR*‐mutant non‐small cell lung cancer (NSCLC).^1^ In 2018, Food and Drug Administration approved dacomitinib in the above setting due to its significantly improved median progression‐free survival (mPFS) over gefitinib (14.7 vs. 9.2 months, *p *< 0.0001) in the ARCHER 1050 study.

However, most studies focused primarily on the later‐line use of dacomitinib (including ARCHER 1009, ARCHER 1028, and BR.26)^2^, ^3^, ^4^ failed primary endpoint. Even in retrospectively grouped patients bearing *EGFR* mutations from ARCHER 1009 and ARCHER 1028,^4^ benefits in mPFS between dacomitinib and erlotinib did not emerge (7.44 vs. 7.44 months, HR 0.46, 95% CI 0.18–1.18; *p *= 0.098). In subgroup analysis for patients harboring *EGFR* mutations from BR.26 study (dacomitinib vs. placebo), dacomitinib slightly improved the PFS from 0.95 to 3.52 months, but the overall survival (OS) remained unchanged (7.23 vs. 7.52 months, HR 0.98, 95% CI 0.67–1.44, *p* = 0.46),^2^ leading to its application in patients who have progressed after 1G TKIs, or after conventional cytotoxic chemotherapy absent of robust evidence.

Notably, due to study design, re‐biopsy difficulty, and blood gene testing availability at that time, these clinical trials did not include mutation status after front‐line TKI treatment, especially for T790M (the key resistance mechanism to 1G/2G TKIs, being detected in more than 50% patients), as a basis for grouping and subgroup analysis. Although dacomitinib efficacy against T790M mutation was observed in a variety of human tumor xenograft models,^5^ effective inhibition of T790M is only possible at clinically unattainable concentrations. On the other hand, in patients who are negative for T790M and other targetable targets (e.g., *MET* [MET proto‐oncogene, receptor tyrosine kinase] mutations) after progression on a front‐line 1G/2G TKI, dacomitinib is theoretically more therapeutically advantageous due to its general inhibition of the entire *EGFR* family.

In this study, we retrospectively analyzed 56 Chinese NSCLC patients harboring *EGFR* mutations progressed on previous therapies in two medical centers, explored the treatment patterns, and evaluated the real‐world efficacy and safety of later‐line dacomitinib utilizing next‐generation sequencing (NGS).

## 2 PATIENTS AND METHODS

### 2.1 Patient eligibility and data collection

Patients were retrospectively collected from the Chinese PLA General Hospital and National Cancer Center/National Clinical Research Center for Cancer/Cancer Hospital, Chinese Academy of Medical Sciences, and Peking Union Medical College. Eligible patients had a histologically confirmed diagnosis of unresectable stage III or stage IV NSCLC harboring NGS‐confirmed *EGFR* mutations. All patients had received standard front‐line treatment (TKI and/or chemotherapy) and progressed before dacomitinib administration. Tumor tissue or cell‐free DNA (cfDNA) from plasma, pleural effusion, or cerebrospinal fluid samples before dacomitinib treatment were tested using targeted NGS, which was performed by qualified third‐party genetic testing companies that had been accredited by the College of American Pathologists (CAP). The NGS was performed as routine care in our study population, and the results were already available before the patients initiated dacomitinib administration. This research was conducted under the Declaration of Helsinki and approved by Research Ethics Board. Because this was a retrospective cohort study, informed consent was waived.

### 2.2 Treatment and efficacy/toxicity evaluation

All patients were treated with later‐line single dacomitinib or combinatory dacomitinib. The starting dose was determined by the physician based on the patient condition. In general, the starting dose was 45 mg for patients with the Eastern Cooperative Oncology Group (ECOG) performance status (PS) of 0 and the weight ≥60 kg; 30 mg for patients with the PS of 1 and the weight <60 kg; 30 mg for patients with the PS of 1 and the weight ≥60 kg, with an increase to 45 mg if the patient could tolerate well; and 15 mg for patients with PS ≥2. Tumor responses assessed according to the Response Evaluation Criteria in Solid Tumors (RECIST 1.1) include complete remission (CR), partial remission (PR), stable disease (SD), and progressive disease (PD). The ORR was defined as the sum of CR + PR ratio, and the DCR was defined as the sum of CR + PR + SD ratio. Toxicity was assessed according to the Common Terminology Criteria for Adverse Events (CTCAE) version 4.0.

### 2.3 Statistical analysis

Categorical variables are reported as numbers and percentages. Survival curves were created using the Kaplan–Meier method and compared using the Log‐rank test. Univariate and multivariate Cox proportional hazards models were used to evaluate the relationships between the various characteristics and OS/PFS, with the results reported as hazard ratios (HRs) and 95% confidence intervals (CIs). The multivariate analysis included significant characteristics from the univariate analyses and characteristics that were considered clinically significant and was performed using the backward likelihood ratio (Backward LR) test. Scatter plots were used to assess correlations and Pearson coefficients were used to assess the strength of correlations. The data cut‐off date was August 15, 2021, when the survival status of the patients was determined. PFS was defined as the time from dacomitinib administration to disease progression or death from any cause, whereas OS was defined as the time from dacomitinib administration to death from any cause. Statistical analyses were performed and pictures were created using GraphPad Prism 8 software (GraphPad Software). Differences were considered statistically significant at two‐sided *p* values of less than 0.05.

## 3 RESULTS

### 3.1 Baseline characteristics

In this retrospective study, 56 *EGFR*‐mutant NSCLC patients with/without brain metastases were treated with later‐line dacomitinib between August 2019 and August 2021. Table 1 demonstrated the demographic, clinicopathologic, and molecular characteristics of this cohort. Nearly 47% of patients were women (26 of 56) and more than 62% were non‐smokers (35 of 56). Ages varied between 31 and 83 years. Almost all (55 of 56) pa tients had adenocarcinoma as the main histologic type of their tumor. Of the 56 patients, 5 had stage III and 45 had stage IV, and 6 patients had undergone resection of early‐stage lung cancer or radical chemoradiotherapy previously and they had recurred as stage IV NSCLC. As to the mutation type, most patients (53.6%) harbored 21L858R, and nearly 30% carried 19del; rare mutations accounted for nearly 20%. Twenty‐three patients (41.1%) had one or more nodules in the brain. Patients had received a median of 3 (range, 2–13) previous lines of therapies before dacomitinib for advanced disease; most patients (33.9%) received dacomitinib as third‐line therapy. The most frequently used TKIs before dacomitinib were osimertinib, gefitinib, and icotinib, respectively. For TKI‐pretreated patients (*n* = 54), most patients (50 patients, 92.6%) were negative for T790M before dacomitinib administration. The ECOG PS of the 56 patients ranged from 0 to 4; 5 (8.9%) patients had a score of 0, 44 (78.6%) had a score of 1, and 7 (12.5%) patient had a score of ≥2.

**TABLE 1:** Baseline characteristics (*n* = 56)

Characteristics	*n* (%)
Median age (range)/year	62 (31–83)
Gender/*n* (%)	
Female	26 (46.4%)
Male	30 (53.6%)
Smoking history/*n* (%)	
Yes	17 (30.4%)
No	35 (62.5%)
Unknown	4 (7.1%)
Histology/*n* (%)	
AC	55 (98.2%)
SCC	1 (1.8%)
Disease stage at the initiation of prior *EGFR*‐TKI/*n* (%)	
IIIB/IIIC^b^	5 (8.9%)
IV	45 (80.4%)
Recurrence after surgical resection or radical chemoradiotherapy	6 (10.7%)
*EGFR* mutation status/*n* (%)	
19del	15 (26.8%)
21L858R	30 (53.6%)
Other rare mutations^c^	11 (19.6%)
Brain metastases/*n* (%)	
Yes	23 (41.1%)
No	33 (58.9%)
Tumor burden/*n* (%)	
≥3 metastatic organs	13 (23.2%)
<3 metastatic organs	43 (76.8%)
Dacomitinib application line^a^/*n* (%)^d^	Median (range): 3 (2–13)
2	16 (28.6%)
3	19 (33.9%)
4	12 (21.4%)
≥5	9 (16.1%)
Previous use of *EGFR*‐TKIs/*n* (%)	
Gefitinib/Elotinib/Icotinib	21/10/20 (37.5%/17.9%/35.7%)
Afatinib	13 (23.2%)
Osimertinib/Almonertinib/Avitinib	30/1/1 (53.6%/1.8%/1.8%)
T790M status before dacomitinib administration	
Positive	52 (92.9%)
Negative	4 (7.1%)
Dacomitinib treatment patterns/*n* (%)	
Single dacomitinib	36 (64.3%)
Dacomitinib + other TKI	9 (16.1)
Dacomitinib + chemotherapy	4 (7.1%)
Dacomitinib + bevazumab	4 (7.1%)
Dacomitinib + other TKI + chemotherapy	1 (1.8%)
Dacomitinib + other TKI + bevazumab	1 (1.8%)
Dacomitinib + bevazumab + chemotherapy	1 (1.8%)
Dacomitinib dosage/*n* (%)^d^	
15 mg	11 (19.6%)
30 mg	32 (57.1%)
45 mg	13 (23.2%)
ECOG PS/*n* (%)	
0	5 (8.9%)
1	44 (78.6%)
≥2	7 (12.5%)

Abbreviations: AC, adenocarcinoma; ECOG PS, Eastern Cooperative Oncology Group performance status.

^a^ Including cytotoxic chemotherapy, immunotherapy, and molecularly targeted agents; SCC, squamous cell carcinoma.

^b^ Patients refused to receive chemoradiotherapy.

^c^ Including G719X, L747P, S768I, and L861Q.

^d^ Percentages might add up to more than 100% due to rounding;

### 3.2 Treatment modality and toxicity/efficacy evaluation

The single dacomitinib application was the main treatment pattern (64.3%), only 35.7% of patients received combinatory dacomitinib treatment (specific combination regimens were displayed in Table 1). A starting dose of 45 mg once daily was given to 13 (23.2%) patients, 30 mg once daily to 32 (57.1%) patients, and 15 mg once daily to the remaining 11 (19.6%) patients. According to the treatment modality of dacomitinib, the whole cohort can be divided into the 1G/2G TKI→dacomitinib cohort (*n* = 25), and 3G TKI→dacomitinib cohort (*n* = 29), and TKI‐naïve cohort (*n* = 2).

**FIGURE 1:**
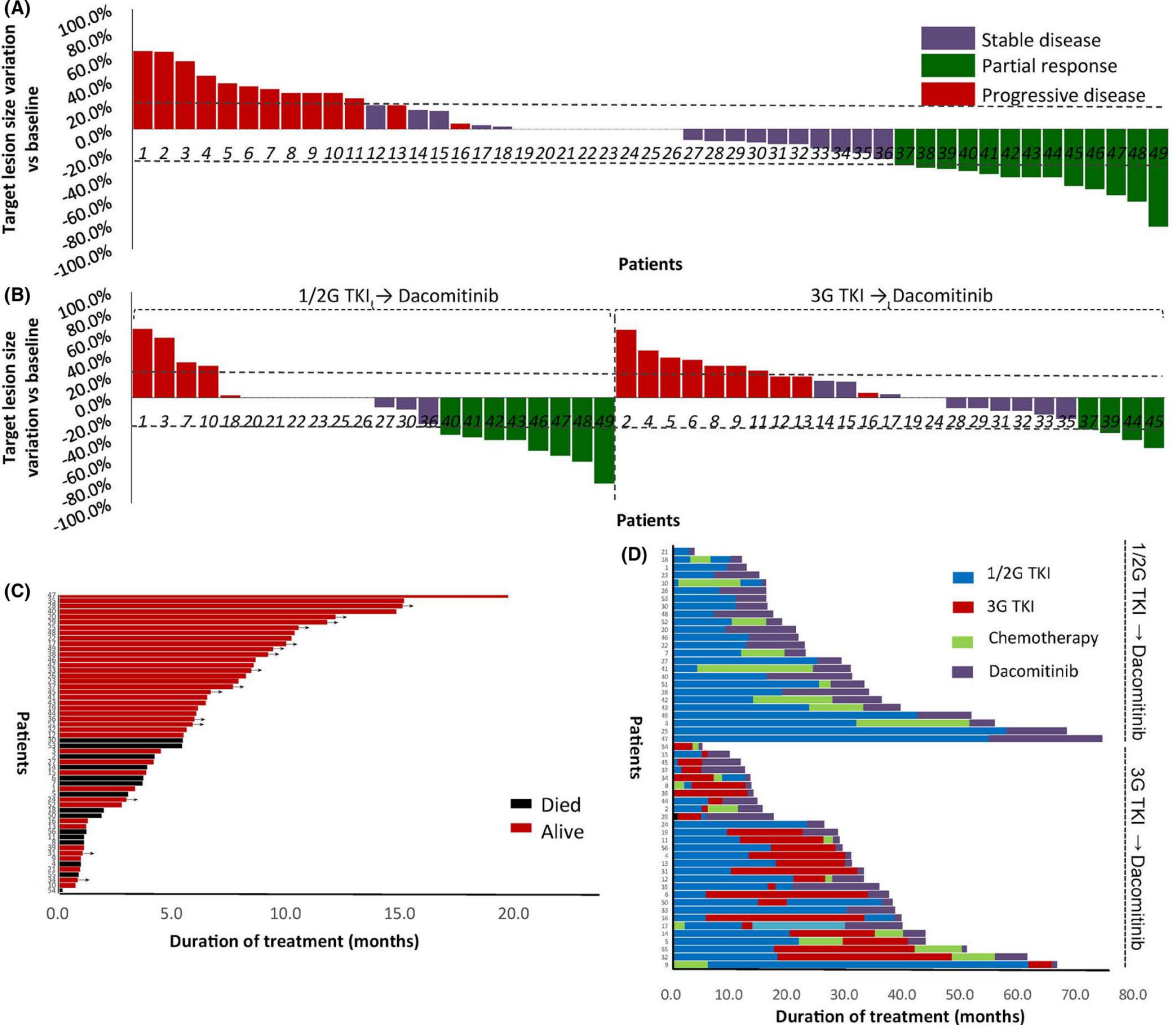
Best change in total target lesion size and duration of treatment by the patient. (A) Waterfall diagram of the evaluable whole cohort (*n* = 49). (B) Waterfall diagram of the 1G/2G TKI→dacomitinib cohort (*n* = 22) and 3G TKI→dacomitinib cohort (*n* = 27). (C) Duration of dacomitinib treatment of the whole cohort (*n* = 56). Red strips indicate patients who remained alive at the time of data cutoff. (D) Overall treatment duration of the 1G/2G TKI→dacomitinib cohort (*n* = 25) and 3G TKI→dacomitinib cohort (*n* = 29). The duration of treatment with dacomitinib is shown in purple, with prior 1G/2G TKI in blue, chemotherapy in green, and osimertinib in red. Dashed lines represent 20% progression (progressive disease) and 30% tumor regression (partial response)

**FIGURE 2:**
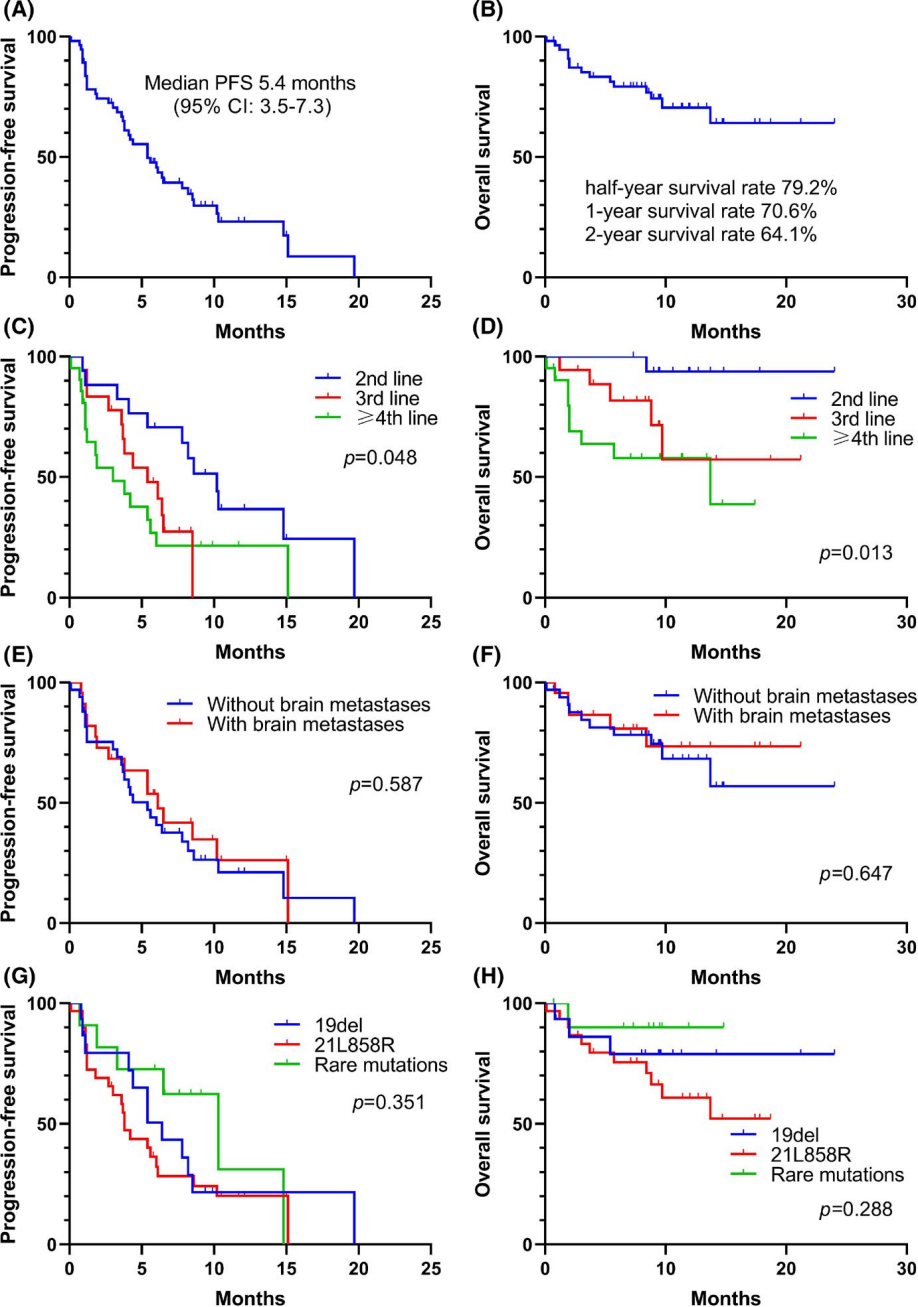
Kaplan–Meier analysis of progression‐free survival and overall survival (*n* = 56). The PFS and OS of the whole cohort were shown in A and B. Different lines of dacomitinib treatment demonstrated statistically different PFS (*p *= 0.048) (C) and OS (*p *= 0.013) (D). No significant effect on PFS (*p *= 0.587) (E) and OS (*p* = 0.647) (F) of dacomitinib regardless of brain metastases. Although no statistical difference was reached, different mutation subtypes demonstrated different PFS (*p *= 0.351) (G) and OS (*p* = 0.288) (H)

Among 49 evaluable patients in the whole cohort, 26.5% (13 patients) achieved PR and 46.9% (23 patients) had SD, with an ORR of 26.5% and an DCR of 73.5% (Figure 1A). Moreover, the ORRs of dacomitinib in the patients treated in second‐line (*n* = 15), third‐line (*n* = 15), fourth‐line (*n* = 9), and ≥5th‐line (*n* = 10) were 40.0%, 33.3%, 22.2%, and 0%, respectively (Figure S1A), and those in patients treated with single dacomitinib and combinatory dacomitinib were 34.4% and 11.8% (Figure S1B). For brain metastases, the ORR of patients with brain metastases was lower than that of patients without brain metastases (16.7% vs. 32.3%) (Figure S1C). As to mutation types, the ORRs of 19del, 21L858R, and rare mutations were 28.6%, 12.5%, and 54.5%, respectively (Figure S1D). In the whole cohort (*n* = 56), the median duration of follow‐up was 9.6 months (95% confidence interval [CI], 8.4–10.8 months). At the data cut‐off date, the PFS was mature in 41 patients and tumors of 15 patients are still under control (Figure 1C), and the mPFS was 5.4 months (95% CI, 3.5–7.3 months) (Figure 2A); the OS was mature in 15 patients and the mOS was unreached, and the half‐year, 1‐year, and 2‐year OS rate were 79.2%, 70.6%, and 64.1%, respectively (Figure 2B).

In the 1G/2G TKI→dacomitinib cohort (*n* = 22) and 3G TKI→dacomitinib cohort (*n* = 25), the ORRs were 36.4% and 16.0%, respectively (Figure 1B); in the TKI‐naïve cohort (*n* = 2), the ORR was 50.0%. In the 1G/2G TKI→dacomitinib cohort (*n* = 25) and 3G TKI→dacomitinib cohort (*n* = 29), the mPFS were 7.8 and 3.8 months, respectively, and the mOS were not reached and 13.7 months, respectively. The swimming diagrams of the above two subgroups are shown in Figure 1D, which also depicted the treatment history of the two groups of patients.

A total of 53 patients (94.6%) had treatment‐related adverse events (TRAEs) and 8 patients (14.3%) had grade 3 or 4 TRAEs, but no grade 5 TRAEs occurred (Table 2).

**TABLE 2:** Treatment‐emergent AEs (*n* = 56)

AEs	G1	G2	G3	G4
Diarrhea	21 (37.5%)	8 (14.3%)	1 (1.8%)	0
Rash	20 (35.7%)	13 (23.3%)	5 (8.9%)	0
Oral mucositis	10 (17.9%)	7 (12.5%)	2 (3.6%)	0
Paronychia	8 (14.3%)	8 (14.3%)	0	0
Dry skin	9 16.1(%)	3 (5.4%)	0	0
Hemorrhinia	1 (1.8%)	1 (1.8%)	0	0
Nausea	3 (5.4%)	1 (1.8%)	0	0
Fatigue	2 (3.6%)	0	0	0
Interstitial pneumonia	0	0	0	1 (1.8%)
Liver dysfunction	0	1 (1.8%)	0	0

Note

Data are *n* (%). AEs, adverse events. There were no grade 5 treatment‐emergent AEs.

### 3.3 Survival comparison

Different lines of dacomitinib treatment demonstrated statistically different PFS (log‐rank *p *= 0.048) (Figure 2C) and OS (log‐rank *p* = 0.013) (Figure 2D), and the PFS of dacomitinib in the patients treated in second‐line (*n* = 17), third‐line (*n* = 18), and ≥4th‐line (*n* = 21) were 10.2, 5.4, and 3.0 months, respectively. Our analysis showed no significant effect on PFS (Log‐rank *p *= 0.587) (Figure 2E) and OS (Log‐rank *p* = 0.647) (Figure 2F) of dacomitinib regardless of brain metastases, reflecting the strong penetrating ability to the blood‐brain barrier and the potent efficacy for central nervous system metastasis. Although no statistical difference was reached, different mutation subtypes demonstrated different PFS (mPFS, 10.3 vs. 6.4 vs. 3.8; *p *= 0.351) (Figure 2G) and OS (1‐year OS rate, 90.0% vs. 79.0% vs. 60.9%; *p* = 0.288) (Figure 2H), with rare mutations (*n* = 11) demonstrating better treatment responses and prognosis, followed by 19del (*n *= 15) and 21L858R (*n* = 30).

Interestingly, when we analyzed the effect of chemotherapy on the efficacy of dacomitinib between the application of the last TKI and dacomitinib, we found that patients who did not receive chemotherapy during the period had a better PFS (*p* = 0.027) than those who did, although not a better OS (*p *= 0.141) (Table 3). In addition, when we compared the efficacy of chemotherapy with dacomitinib after the last TKI treatment, a trend of better PFS (mPFS, 6.5 vs. 5.0 months; *p* = 0.265) and OS (mOS, not reached vs. 13.7 months; *p *= 0.130) of dacomitinib was revealed, although differences were not significant (Table 3). We also found a significant shortening of both mPFS (*p *= 0.036) and mOS (*p *= 0.045) when the duration of the interval between the last TKI and dacomitinib exceeded 4 months. Moreover, a better OS (not reached vs. 13.7 months, HR = 0.333, 95% CI 0.106–0.978; *p *= 0.048) but not PFS (Figure S2D) (7.8 vs. 3.8 months, HR = 0.683, 95% CI 0.365–1.277; *p *= 0.232) of 1G/2G TKI→dacomitinib cohort than 3G TKI→dacomitinib cohort was indicated (Table 3).

**TABLE 3:** Univariate and multivariate cox regression analysis

Variables^a^	*n*	PFS				OS			
		Median (months)	HR	95% CI	*p* value	Average^b^ (months)	HR	95% CI	*p* value
Univariate cox regression analysis									
Age									
≤60/>60 years	25/31	6.5/5.4	0.852	0.454–1.598	0.617	18.7/14.0	0.892	0.316–2.517	0.829
Gender									
Male/female	30/26	5.6/5.4	0.837	0.441–1.587	0.585	18.1/13.2	0.937	0.339–2.592	0.900
Smoking									
No/yes + unknown	35/21	6.1/4.4	0.514	0.276–0.958	**0.036**	19.5/13.0	0.367	0.131–0.978	**0.049**
ECOG PS									
0–1/2–4	49/7	5.4/6.0	0.880	0.310–2.491	0.809	18.1/8.4	0.427	0.118–1.537	0.193
Disease stage									
III + IV/recurrence	50/6	5.6/3.6	0.913	0.332–2.584	0.864	17.7/12/1	0.996	0.224–4.426	0.996
Tumor burden									
<3/≥3 metastatic organs	43/13	6.1/3.3	0.617	0.299–1.276	0.193	18.3/9.8	0.519	0.176–1.534	0.235
*EGFR* mutation subtypes									
19del/21L858R	15/30	6.4/3.8	0.778	0.377–1.608	0.498	19.5/12.9	0.545	0.152–1.957	0.352
19del/rare mutations	15/11	6.4/10.3	1.524	0.561–4.142	0.409	19.5/13.5	2.115	0.220–20.362	0.517
21L858R/rare mutations	30/11	3.8/10.3	1.904	0.770–4.705	0.163	12.9/13.5	3.856	0.496–29.957	0.197
Brain metastases									
No/yes	33/23	5.4/6.1	1.192	0.628–2.263	0.591	16.7/16.7	1.285	0.438–3.770	0.648
Dacomitinib combination therapy									
No/yes	36/20	5.4/5.6	1.058	0.544–2.057	0.868	16.2/16.0	2.402	0.678–8.513	0.175
Initial dosage of dacomitinib									
45/30 mg	13/32	6.1/5.6	1.025	0.455–2.309	0.952	15.0/17.5	0.789	0.213–2.916	0.722
45/15 mg	13/11	6.1/3.8	0.746	0.276–2.018	0.746	15.0/14.2	1.069	0.201–5.672	0.938
30/15 mg	32/11	5.6/3.8	0.748	0.342–1.639	0.469	17.5/14.2	1.099	0.296–4.087	0.888
Treatment line of dacomitinib									
2/3	17/18	8.2/4.2	0.335	0.127–0.880	**0.026**	21.3/13.1	0.140	0.016–0.987	**0.047**
3/≥4	18/21	4.2/4.1	0.726	0.343–1.537	0.403	13.1/14.1	0.525	0.176–1.570	0.249
2/≥4	17/21	8.2/4.1	0.439	0.201–0.962	**0.040**	21.3/14.1	0.090	0.011–0.713	**0.023**
Time on treatment of first *EGFR*‐TKI									
≦6/>6 months	13/41	5.4/5.4	1.136	0.519–2.486	0.750	11.2/17.9	0.799	0.254–2.511	0.700
≦12/>12 months	29/25	5.4/5.6	0.804	0.423–1.525	0.504	13.0/18.8	0.665	0.236–1.877	0.441
≦20/>20 months	42/12	5.4/5.4	0.684	0.314–1.490	0.339	14.9/20.8	0.466	0.105–2.069	0.316
Interval between last *EGFR*‐TKI and dacomitinib									
≦4/>4 months	18/36	8.2/3.8	0.497	0.259–0.956	**0.036**	23.1/13.1	0.372	0.710–0.965	**0.045**
≦6/>6 months	23/31	8.6/3.7	0.502	0.261–0.968	**0.040**	22.2/12.5	0.443	0.160–1.227	0.117
≦8/>8 months	24/30	8.6/3.7	0.527	0.267–1.040	0.065	22.3/12.3	0.433	0.156–1.199	0.107
*EGFR*‐TKI generation followed by dacomitinib									
1 or 2G/3G	25/29	7.8/3.8	0.683	0.365–1.277	0.232	20.4/12.2	0.333	0.106–0.978	**0.048**
Chemotherapy between last *EGFR*‐TKI and dacomitinib									
No/yes	36/18	6.5/3.8	0.465	0.236–0.916	**0.027**	19.2/12.7	0.466	0.168–1.288	0.141
Treatment after last *EGFR*‐TKI									
Chemotherapy/dacomitinib	18/36	5.0/6.5	1.627	0.853–3.103	0.265	9.55/7.37	2.140	0.702–6.525	0.130
Multivariate cox regression analysis									
Smoking									
No/yes + unknown	35/21						0.270	0.093–0.785	**0.016**
Treatment line of dacomitinib									
2/3/≥4	17/18/21								
Interval between last *EGFR*‐TKI and dacomitinib									
≦4/>4 months	18/36								
*EGFR*‐TKI generation followed by dacomitinib									
1 or 2G/3G	25/29						0.238	0.073–0.776	**0.017**
Chemotherapy between last *EGFR*‐TKI and dacomitinib									
No/yes	34/20		0.463	0.235–0.91	**0.026**				

Bold values represent the statistical significance.

Abbreviations: 1G, first generation; 2G, second generation; 3G, third generation; CI, confidence interval; ECOG PS, Eastern Cooperative Oncology Group performance status; *EGFR*‐TKI, epidermal growth factor receptor tyrosine kinase inhibitor; HR, hazard ratio; OS, overall survival; PFS, progression‐free survival.

^a^ Set variables behind “/” as reference.

^b^ Since the median overall survival was not reached, the average was used instead.

### 3.4 Survival analysis

Similar to the results from Kaplan–Meier method, univariate regression analysis also revealed that smoking, line of dacomitinib, and interval between last TKI and dacomitinib were associated with PFS and OS; chemotherapy between last TKI and dacomitinib, and TKI generation followed by dacomitinib were respectively associated with PFS and OS. Multivariate analysis indicated that PFS was only independently predicted by chemotherapy between last *EGFR*‐TKI and dacomitinib (HR 0.463; 95% CI: 0.235–0.910; *p *= 0.026) (Table 3) and OS was independently predicted by smoking (HR 0.270; 95% CI 0.093–0.785; *p *= 0.016) and TKI generation followed by dacomitinib (HR 0.238; 95% CI 0.073–0.776; *p *= 0.017).

### 3.5 Correlation analyses

**FIGURE 3:**
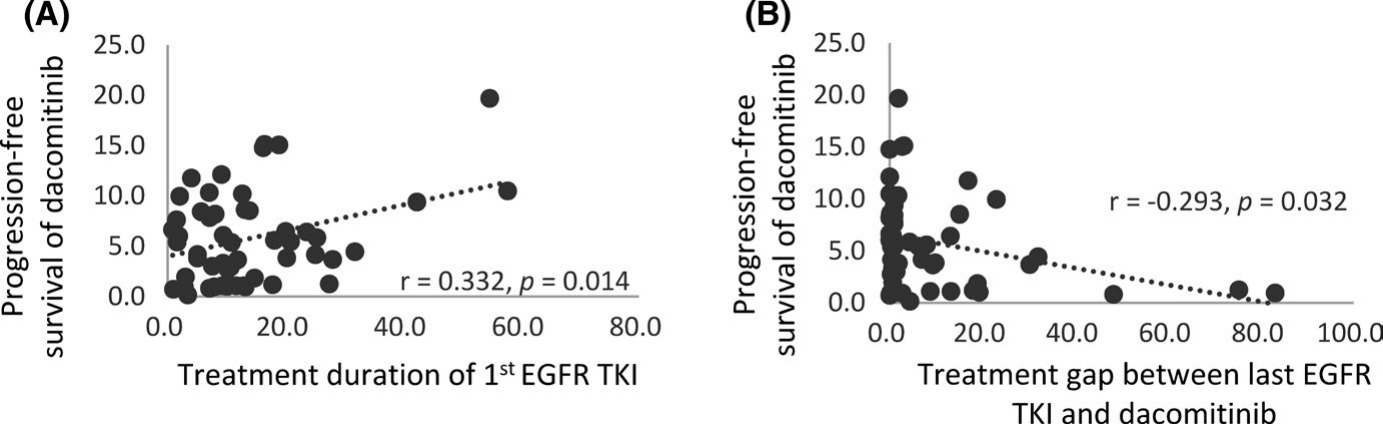
Correlation analyses. A scatter plot analysis of the PFS of dacomitinib between treatment duration of first TKI and interval between last TKI and dacomitinib is shown in A and B

A scatter plot analysis of the PFS of dacomitinib between treatment duration of first TKI and interval between last TKI and dacomitinib is shown in Figure 3(A,B). The PFS of dacomitinib was positively correlated with treatment duration of first TKI (*r* = 0.332, *p *= 0.014) and negatively correlated with interval between last TKI and dacomitinib (*r* = −0.293, *p *= 0.032).

Correlations with the treatment duration of last TKI, the interval between first TKI and dacomitinib, and interval regimen number were also examined and none were found to be associated with the efficacy of dacomitinib administration (data not shown).

## 4 DISCUSSION

This is one of the largest real‐world studies that evaluated the efficacy of dacomitinib for patients with advanced NSCLC harboring *EGFR* mutations in later‐line settings. At the time of analysis, the median duration of follow‐up was 9.6 months (range, 8.4–10.8), the mPFS was 5.4 months (95% CI, 3.5–7.3 months), and the half‐year, 1‐year, and 2‐year OS rate were 79.2%, 70.6%, and 64.1%, respectively. The ORR and DCR were 26.5% and 73.5% in the whole cohort, respectively. Multivariate analysis indicated chemotherapy between last *EGFR*‐TKI and dacomitinib negatively affect PFS, and smoking and third‐generation *EGFR*‐TKI followed by dacomitinib negatively affect OS. Correlation analysis revealed PFS of dacomitinib was positively correlated with treatment duration of first TKI and negatively correlated with interval between last TKI and dacomitinib. Our results show that dacomitinib is active in advanced NSCLC patients harboring *EGFR* mutations in later‐line settings.

Currently, chemotherapy (with or without anti‐vascular drugs or immunotherapy) is recommended for patients who have been resistant to previous TKI(s) and harbor no secondary targetable genes^6^, ^7^, ^8^; however, post‐TKI chemotherapy has only mediocre efficacy. Therefore, patients harboring sensitizing *EGFR* mutations and progressed on previous TKI therapy have an unsatisfied medical need.

For patients after 1G/2G TKI resistance, in the 1G/2G TKI→dacomitinib cohort of our study, an ORR of 36.4% was obtained, with an mPFS of up to 7.8 months, while mOS has not been achieved; however, the results of the classic AURA3 study showed that for patients treated with conventional chemotherapy regimens after the failure of first‐line *EGFR*‐TKI therapy, the mPFS and mOS was only 4.4 and 22.5 months respectively, with an ORR of 31.0%.^9^ This suggests that the application of dacomitinib after 1G/2G TKI progression may be no worse than or even better than chemotherapy. In the phase III study BR.26,^2^ the efficacy of dacomitinib versus placebo was assessed in patients progressing on conventional chemotherapy and at least one previous first‐generation TKI (erlotinib or gefitinib). In re‐grouped patients harboring *EGFR* mutations, dacomitinib obtained an ORR of 11.4%, and slightly improved the mPFS from 0.95 to 3.52 months, but did not prolong the mOS (7.52 vs. 7.23 months). We speculate that the reasons for the markedly improved ORR and prolonged PFS in our study include the following: (1). Clear genetic testing guidance. All patients in our study underwent genetic testing before dacomitinib, and the vast majority (52/56) of them were T790M‐negative patients. As shown in our study, three of the four T790M‐positive patients showed *de novo* resistance to dacomitinib, and only 1 patient got SD as best response; (2). The majority of patients in the 1G/2G TKI→dacomitinib cohort (16/25) did not receive chemotherapy before dacomitinib. As found in our study, the absence of chemotherapy between front‐line TKI and dacomitinib resulted in a longer PFS.

For patients after 3G TKI (mostly osimertinib) resistance, in the 3G TKI→dacomitinib cohort of our study, an ORR of 16.0% was achieved, with an mPFS of 3.8 months and mOS of 13.7 months.^10^ This was very close to the study data reported by White et al.,^11^ in which the patients with advanced *EGFR*‐mutant lung cancer received platinum doublet chemotherapy regimens after progression on osimertinib got a median time on treatment of 3.9 months (95% CI 1.9–7.8 months) and OS of 12.8 months (95% CI 6.9–19.5 months), indicating a comparable efficacy of later‐line dacomitinib and chemotherapy.

When we compared the efficacy of chemotherapy with dacomitinib head‐to‐head after the last TKI treatment in our whole cohort, a trend of better ORR (29.4% vs. 12.5%, *p* = 0.192), PFS (mPFS, 6.5 vs. 5.0 months; *p* = 0.103) and OS (mOS, not reached vs. 13.7 months; *p *= 0.130) of dacomitinib was revealed, although differences were not significant. Our study provides additional evidence for the use of dacomitinib in patients after progression on previous TKI, especially in the circumstances where additional anti‐vascular agents or immunotherapy did not bring additional PFS and OS benefit compared to single chemotherapy.^11^

An interesting phenomenon we found was that 1G/2G TKI→dacomitinib group had a trend for better ORR (36.4% vs. 16%, *p* = 0.211), PFS (7.8 vs. 3.8 months, *p* = 0.232) and OS (not reached vs. 13.7 months, *p *= 0.048) than 3G TKI→dacomitinib cohort. This is also supported by the identification of 3G *EGFR*‐TKI followed by dacomitinib as a poor independent predictor of OS in multivariate analysis. We speculate that there are two reasons for this result. Firstly, most patients in the 3G TKI→dacomitinib group had received 1G/2G TKI treatment before 3G TKI treatment. In addition, there is evidence that, compared with patients who are resistant after 1G/2G TKI, patients who are resistant after 3G TKI osimertinib have more off‐target resistance mechanisms outside *EGFR* that dacomitinib cannot overcome.^12^

Currently, classical *EGFR* mutations (19DEL and 21L858R) are highly sensitive to different‐generation *EGFR*‐TKIs; however, uncommon mutations are less sensitive, with reported less satisfying response rates and survival in many studies.^13^, ^14^, ^15^, ^16^, ^17^, ^18^ A combined post hoc analysis of LUX‐Lung 2, LUX‐Lung 3, and LUX‐Lung 6 demonstrated that 2G *EGFR*‐TKI afatinib was active (mostly in first‐line setting) in NSCLC patients that harbored certain types of uncommon *EGFR* mutations, especially for G719X, L861Q, and S768I, with an ORR of 71.1%, an mPFS of 10.7 months, and an mOS of 19.4 months, respectively.^19^ So far, there is a lack of data on another 2G TKI dacomitinib for the treatment of NSCLC patients with rare *EGFR* mutations. A total of 11 patients (mostly after progression with afatinib) carrying rare mutations receiving later‐line dacomitinib were included in our study. Unexpectedly, those patients obtained an ORR of 54.5%, an mPFS of 10.3 months, and a 1‐year OS rate of 90.0%, which were even better than those of 19del and 21L858R (ORR: 54.5% vs. 28.6% vs. 11.1%, *p *= 0.018; mPFS: 10.3 vs. 6.4 vs. 3.8 months, *p *= 0.351; 1‐year OS rate: 90.0% vs. 79.0% vs. 60.9%; *p* = 0.288), although no significant statistical difference was reached for PFS and OS. Our study demonstrated that the effect of later‐line dacomitinib was active for patients with NSCLC harboring rare mutations, and it also indicated the feasibility of dacomitinib application after afatinib progression for this subset of patients.

Recently, Peng et al.^20^ has shown that dacomitinib has potent efficacy for central nervous system (CNS) metastasis in *EGFR*‐positive NSCLC. In our study, there were no significant differences in PFS and OS between patients with and without brain metastases, reflecting the effectiveness of dacomitinib in patients carrying brain metastases in later‐line settings. On the other hand, a total of 15 of all 22 patients with brain metastases were symptomatic, of which 9 patients (60.0%) had symptom relief. In addition, only 3 (13.6%) of these 22 patients with brain metastases progressed again due to brain progression.

According to the ARCHER 1050 study,^21^ the OS benefit was maintained in those patients who had a stepwise dose reduction of dacomitinib (from 45 to 30 or 15 mg per day). In our study, there was no significant difference in ORR (18.2% vs. 28.0% vs. 30.8%), mPFS (3.8 vs. 5.6 vs. 6.1 months, *p *= 0.762), and 1‐year OS rate (76.2% vs. 67.2 vs. 73.8%, *p* = 0.965) in 15, 30, and 45 mg group, respectively. However, due to the limited size of the study, no definite conclusions could be drawn.

First, the study was undersized, resulting in potentially compromised results from the multivariate analysis. Therefore, we included not only significant characteristics from the univariate analyses but also characteristics that were considered clinically significant. Second, the landscape of lung cancer treatment is now changing rapidly, with fewer patients using the 1G TKIs as the 3G TKI osimertinib enters the first‐line recommendation. However, considering factors such as the huge base of lung cancer patients worldwide, the health economics advantage of the 1G TKIs, the disadvantage of osimertinib for Asian populations, etc., studies on the “1+3+2” model or the “1+2” model are still of clinical significance. Last but not least, this was a single‐arm small study, which might have led to patient selection bias. Thus, our data should be interpreted cautiously. Furthermore, resistance mechanisms of dacomitinib were not investigated.

In conclusion, this real‐world study has shown that dacomitinib is active and well‐tolerated in NSCLC patients harboring different *EGFR* mutations in later‐line settings, even for those with brain metastases. Patients who benefited more from the first TKI were more likely to benefit from dacomitinib. Earlier application of dacomitinib after front‐line TKI resistance may be considered, but more clinical studies need to be conducted to confirm this conclusion.

## CONFLICT OF INTEREST

All authors have completed the ICMJE uniform disclosure form. The authors have no conflicts of interest to declare.

## AUTHOR CONTRIBUTION

HSL, JYZ, XY, HYX, XZH, PYX, and YW have made substantial contributions to this work.

## ETHICAL STATEMENT

The authors are accountable for all aspects of the work in ensuring that questions related to the accuracy or integrity of any part of the work are appropriately investigated and resolved. All procedures performed in studies involving human participants were in accordance with the ethical standards of the institutional and/or national research committee(s) and with the Helsinki Declaration (as revised in 2013).

## Supporting information

Fig S1

## Data Availability

The data that support the findings of this study are available from the corresponding author upon reasonable request.
