# Supplementary material for: A real‐world study of dacomitinib in later‐line settings for advanced non‐small cell lung cancer patients harboring EGFR mutations
Source: Cancer Med. 2022 Jan 12;11(4):1026–36. doi: 10.1002/cam4.4495 (PMC8855913; doi:10.1002/cam4.4495)
Supplement: Supplementary file 1 — Fig S1 [file CAM4-11-1026-s001.pptx]

## Slide 1
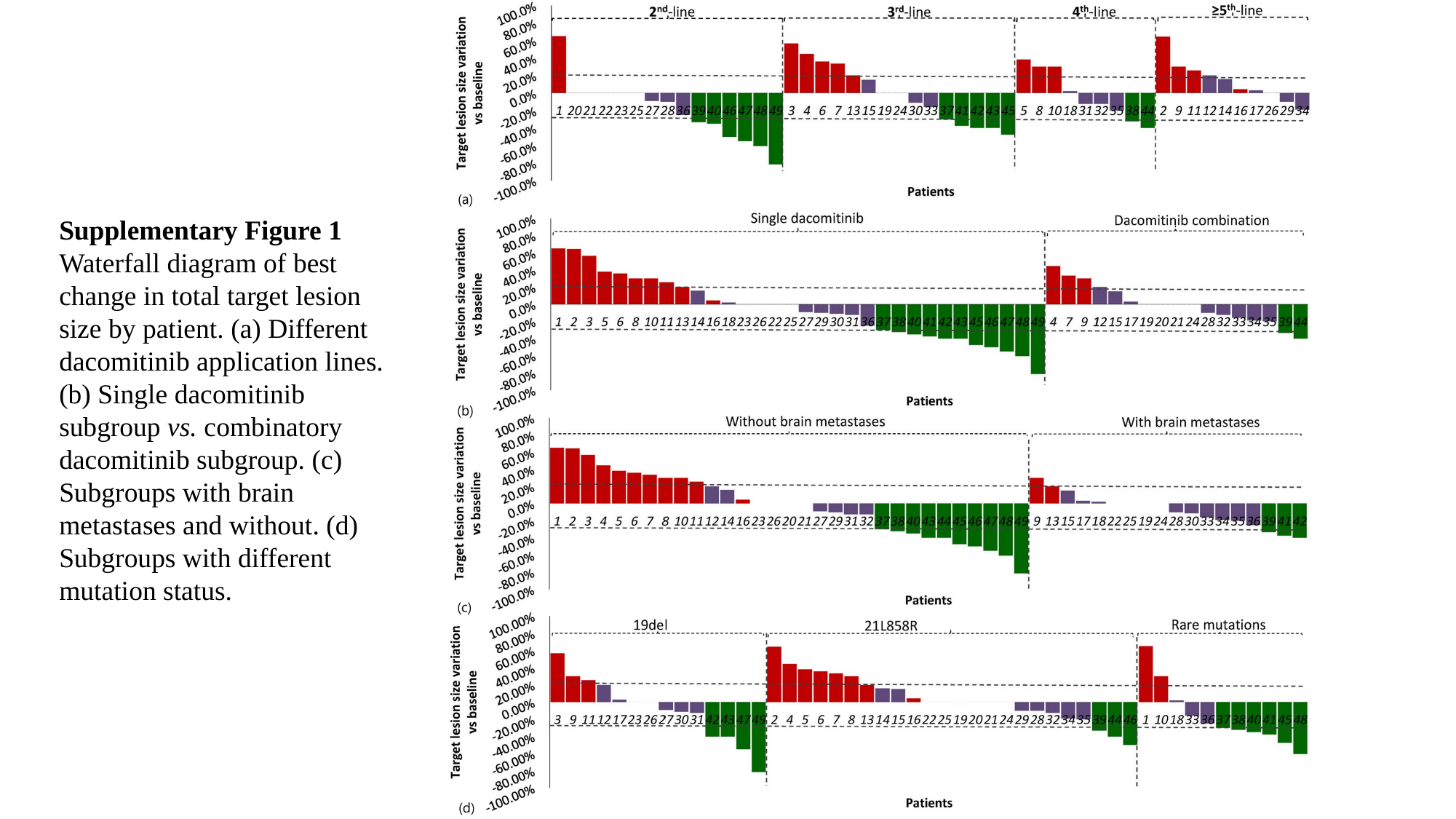

Supplementary Figure 1
Waterfall diagram of best change in total target lesion size by patient. (a) Different dacomitinib application lines. (b) Single dacomitinib subgroup vs. combinatory dacomitinib subgroup. (c) Subgroups with brain metastases and without. (d) Subgroups with different mutation status.
